# Seed coordinates of a new COMS‐like 24 mm plaque verified using the FARO Edge

**DOI:** 10.1120/jacmp.v16i6.5721

**Published:** 2015-11-08

**Authors:** Sarah E. McCauley Cutsinger, Keith M. Furutani, Renae M. Forsman, Stephen M. Corner

**Affiliations:** ^1^ Division of Medical Physics Mayo Clinic Rochester MN; ^2^ Division of Engineering Mayo Clinic Rochester MN

**Keywords:** eye plaque brachytherapy, episcleral plaque, COMS

## Abstract

A 24 mm COMS‐like eye plaque was developed to meet the treatment needs of our eye plaque brachytherapy practice. As part of commissioning, it was necessary to determine the new plaque's seed coordinates. The FARO Edge, a commercially available measurement arm, was chosen for this purpose. In order to validate the FARO Edge method, it was first used to measure the seed marker coordinates in the silastic molds for the standard 10, 18, and 20 mm COMS plaques, and the results were compared with the standard published Task Group 129 coordinates by a nonlinear least squares match in MATLAB version R2013a. All measured coordinates were within 0.60 mm, and root mean square deviation was 0.12, 0.23, and 0.35 mm for the 10, 18, and 20 mm molds, respectively. The FARO Edge was then used to measure the seed marker locations in the new 24 mm silastic mold. Those values were compared to the manufacturing specification coordinates and were found to demonstrate good agreement, with a maximum deviation of 0.56 mm and a root mean square deviation of 0.37 mm. The FARO Edge is deemed to be a reliable method for determining seed coordinates for COMS silastics, and the seed coordinates for the new 24 mm plaque are presented.

PACS number: 87.53.Jw

## I. INTRODUCTION

In the mid‐1980s, the Collaborative Ocular Melanoma Study (COMS) was formed to investigate, in part, differences in survival and quality of life outcomes between treatment of choroidal melanoma by enucleation or use of episclearal plaque brachytherapy. The medium tumor trial was originally constrained to tumors from 3 to 8 mm in height and 16 mm or less in basal diameter.^(1)^ With the recommendation that a 2 mm tumor‐free perimeter be maintained around the tumor for plaque coverage, the largest plaque required at that time was 20 mm.^(2)^

The COMS has now closed, having demonstrated equal survival in both brachytherapy and enucleation patients, and better visual function within the first two years post‐treatment in those treated with brachytherapy.^3^, ^4^ Partly as a result of this, plaque brachytherapy is an accepted standard treatment for medium‐sized choroidal melanoma.

The 12 to 20 mm plaques and silastic seed carriers were designed for the COMS at The Mayo Clinic.^(5)^ Manufacture of these pieces of equipment was, and is, done at Trachsel Dental Studio, Rochester, MN.^(6)^ In more recent years, the 10 mm plaque and 22 mm plaque, as well as their respective seed carriers, were designed and put into manufacture.

There is now an interest in and willingness to treat tumors larger than the original 16 mm basal diameter limit, as evidenced by the creation and use of the 22 mm plaque, as well as the availability of other large plaques from Eye Physics, LLC and Eckert & Ziegler BEBIG. At the request of ophthalmology and radiation oncology staff at Mayo, it was undertaken to develop a COMS‐like 24 mm diameter plaque and corresponding seed carrier, and perform the necessary physical and dosimetric measurements to commission it for clinical use. The scope of this article is limited to the relevant physical measurements.

## II. MATERIALS AND METHODS

**Figure 1:**
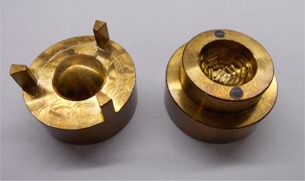
24 mm silastic mold.

**Figure 2:**
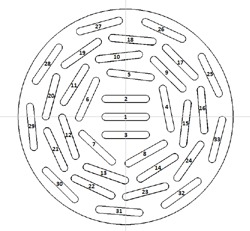
Diagram of the 24 mm seed carrier, as viewed from the concave side. The x‐ and y‐axes are labeled on their positive axes, and z is positive out of the page, with the origin lying at the inner sclera along an axis passing through the center of the eye and the center of seed marker 1.

The new 24 mm silastic mold is shown in Fig. 1, and the diagram of the seed carrier it produces, as viewed from the concave side, is shown in Fig. 2. The new mold's design and construction are modeled after the original COMS silastic molds. The seed markers are 5.3 mm long, 0.8 mm wide, and 1.0 mm in height, which is measured from the concave surface of the seed assembly piece to the top face of the seed markers themselves. The seed marker height does differ slightly from that of the original COMS, which is 1.25 mm, but allows for greater certainty in seed position within the final silastic product. The design of the central portion is similar to that of the 20 mm plaque, with one additional outer ring of nine seeds, bringing the total number of seed slots to 33. Table 1 lists the specification coordinates for the seed centers and seed ends for the 24 mm silastic mold.

**Figure 3:**
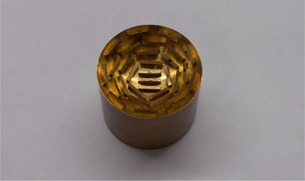
24 mm mold seed assembly piece.

Regardless of which plaque size it corresponds to, each silastic mold easily separates into three pieces. The two larger pieces form the housing for the central portion — the seed assembly. It is the seed assembly which defines where the seed slots will be in the final silastic product. Figure 3 shows the 24 mm mold's seed assembly piece separate from its housing.

Determination of expected seed geometry within a silastic insert requires that measurements be made of either a silastic seed carrier itself or of the appropriate seed assembly piece from the silastic mold. Due to its deformable nature, measurements of silastic have largely been constrained to use of radiographic or photographic methods. The rigidity of the seed assembly is an advantage, then, in enabling physical measurements. However, the curvature of its concave aspect and the close spacing of the seed markers make use of typical measurement devices, such as calipers, somewhat difficult.

**Figure 4:**
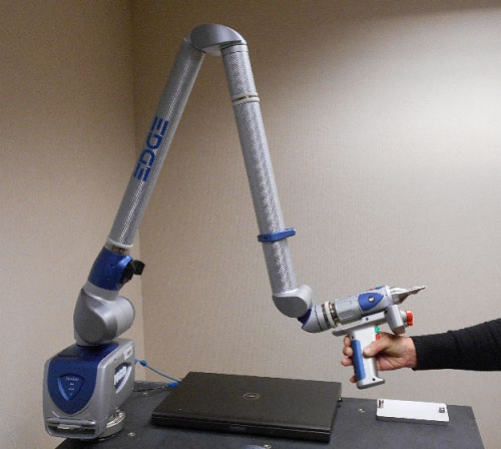
The FARO Edge measurement arm.

To alleviate some of these difficulties, the FARO Edge (pictured in Fig. 4) was chosen to be used to measure the COMS silastic mold seed assemblies. The FARO Edge (FARO Technologies UK Ltd, Coventry, UK) is a measurement arm with three articulating joints that allow for localizing in three dimensions. A small point probe may be fitted to the end of the arm which is suitable for use on the scale of the COMS silastic molds. Coordinates at the tip of the point probe may be recorded in the FARO Edge software system at the user's discretion by the press of a button. Within the range of use, the FARO Edge is specified by the manufacturer as having an accuracy of ±0.034mm and a reproducibility of 0.024 mm.^(7)^

**Table 1:** 24 mm plaque specification coordinates rendered in the TG‐129 coordinate system, as illustrated in Fig. 2

	*Seed Center Coordinates (mm)*			*Seed End Coordinates (mm)*					
*Seed #*	*x*	*y*	*z*	$x_{1}$	$y_{1}$	$z_{1}$	$x_{2}$	$y_{2}$	$z_{2}$
*24 mm plaque*									
1	0.00	0.00	−2.40	2.65	0.00	−2.40	−2.65	0.00	−2.40
2	0.00	1.94	−2.26	2.65	1.94	−2.26	−2.65	1.94	−2.26
3	0.00	−1.94	−2.26	−2.65	−1.94	−2.26	2.65	−1.94	−2.26
4	4.42	0.86	−1.64	4.92	−1.74	−1.64	3.91	3.46	−1.64
5	0.55	4.47	−1.64	3.18	4.14	−1.64	−2.08	4.79	−1.64
6	−4.08	1.90	−1.64	−2.96	4.30	−1.64	−5.20	−0.50	−1.64
7	−3.07	−3.29	−1.64	−5.01	−1.48	−1.64	−1.13	−5.10	−1.64
8	2.18	−3.94	−1.64	−0.14	−5.22	−1.64	4.50	−2.65	−1.64
9	4.65	4.49	−0.77	6.49	2.59	−0.77	2.81	6.40	−0.77
10	−0.61	6.44	−0.77	2.03	6.69	−0.77	−3.25	6.19	−0.77
11	−5.42	3.54	−0.77	−3.97	5.76	−0.77	−6.87	1.32	−0.77
12	−6.14	−2.03	−0.77	−6.97	0.49	−0.77	−5.31	−4.55	−0.77
13	−2.24	−6.07	−0.77	−4.73	−5.15	−0.77	0.24	−6.99	−0.77
14	3.35	−5.54	−0.77	1.08	−6.91	−0.77	5.61	−4.17	−0.77
15	6.41	−0.84	−0.77	6.07	−3.46	−0.77	6.76	1.79	−0.77
16	8.25	0.72	0.40	8.48	−1.92	0.40	8.02	3.36	0.40
17	5.86	5.86	0.40	7.73	3.98	0.40	3.98	7.73	0.40
18	0.72	8.25	0.40	3.36	8.02	0.40	−1.92	8.48	0.40
19	−4.75	6.78	0.40	−2.58	8.30	0.40	−6.92	5.26	0.40
20	−8.00	2.14	0.40	−7.31	4.70	0.40	−8.69	−0.42	0.40
21	−7.51	−3.50	0.40	−8.63	−1.10	0.40	−6.39	−5.90	0.40
22	−3.50	−7.51	0.40	−5.90	−6.39	0.40	−1.10	−8.63	0.40
23	2.14	−8.00	0.40	−0.42	−8.69	0.40	4.70	−7.31	0.40
24	6.78	−4.75	0.40	5.26	−6.92	0.40	8.30	−2.58	0.40
25	9.12	4.45	2.14	10.28	2.06	2.14	7.95	6.83	2.14
26	4.13	9.27	2.14	6.55	8.19	2.14	1.70	10.34	2.14
27	−2.80	9.75	2.14	−0.25	10.48	2.14	−5.34	9.02	2.14
28	−8.41	5.67	2.14	−6.93	7.87	2.14	−9.89	3.48	2.14
29	−10.09	−1.06	2.14	−10.36	1.58	2.14	−9.81	−3.70	2.14
30	−7.05	−7.30	2.14	−8.95	−5.46	2.14	−5.14	−9.14	2.14
31	−0.71	−10.12	2.14	−3.35	−9.93	2.14	1.94	−10.30	2.14
32	5.96	−8.21	2.14	3.82	−9.76	2.14	8.11	−6.65	2.14
33	9.84	−2.45	2.14	9.20	−5.03	2.14	10.48	0.12	2.14

In order to take seed coordinate data, the seed assembly pieces were removed from the 10, 18, 20, and 24 mm silastic molds and were mounted on the FARO Edge table. The point probe of the FARO Edge was then used to collect multiple datasets of the coordinates at the top surface of the tips of every seed marker within each seed assembly piece.

Measured datasets were averaged together by plaque size. The coordinates were then corrected to account for the fact that the seed markers were measured at their top surfaces rather than at the height of the seed marker center. These corrections assumed the idealized radius of curvature of the concave side of silastic inserts of 12.3 mm and a 1 mm thickness of silastic to the bottom of the seed groove, as specified in the report of Task Group 129.^(8)^ Corrections were fairly minor and tended to impact only larger plaque sizes, due to the more extreme difference in angle of the seed markers on outer rings compared to those in more central rings.

In order to compare the measured data to standard coordinates, the TG‐129 end‐point coordinates were modified only to reflect the 5.3 mm length of the seed markers within the molds, rather than a length of 4.5 mm as specified within the Task Group report. Indeed, measurements performed with digital calipers showed seed marker length to be 5.29 mm ± 0.04 mm. The corrected end‐point measurement data for the 10, 18 and 20 mm molds were then fit to these modified TG‐129 seed coordinates using a nonlinear least squares method in MATLAB (MathWorks, Inc. Natick, MA). Specifically, the lsqnonlin function in MATLAB was applied to an equation of the form:

(1) f=X−A[Y−B]

where *X* is the matrix of TG‐129 or specification end point coordinates of the silastic insert in question, *Y* is the matrix of the FARO‐measured seed end points, and *A* and *B* are rotation and translation matrices, respectively. The translation matrix accounts for the arbitrary origin in the measured FARO data, whereas the rotation matrix corrects for any nonalignment of the coordinate system with respect to the seed assembly piece during measurement as compared to the alignment of the coordinate system with respect to the seed locations in the standard datasets.

The lsqnonlin solver in MATLAB seeks to minimize f through iterative adjustment of A and B. Once the minimum value of f was achieved, the seed coordinates of the matrix equal to A[Y‐B] became the fitted measured dataset used in final comparison to the standard datasets.

This same method was then used for the new 24 mm mold where Y is, as before, the matrix of corrected end‐point FARO Arm measurements, but the matrix X was instead comprised of the manufacturing specification coordinates.

Since all FARO Edge data, save that of the 24 mm plaque, were matched to TG‐129 coordinates, all coordinates are presented in the same coordinate system as TG‐129, detailed in Fig. 2, for clarity.

## III. RESULTS & DISCUSSION

Measured seed end coordinates for all four plaque sizes are listed in Table 2, along with their deviation from the modified TG‐129 or specification coordinates. Where relevant, the seed numbering from TG‐129 is given, as well. All fitted coordinates were within 0.6 mm of the relevant standards. Root mean square error values between the measured data and modified TG‐129 standard or specification values were 0.12 mm, 0.23 mm, 0.35 mm, and 0.37 for the 10, 18, 20, and 24 mm plaques, respectively. The largest disagreement across all four plaques was 0.56 mm.

**Figure 5:**
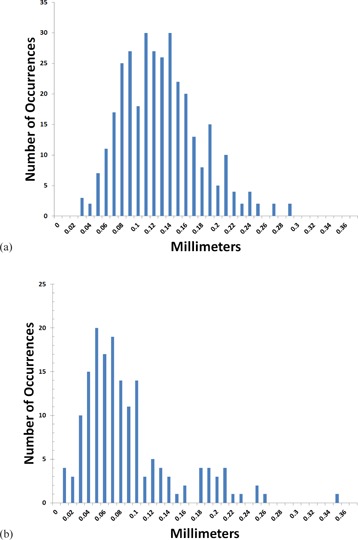
Distribution (a) of X and Y coordinate SDs from the mean over all datasets; distribution (b) of Z coordinate SDs from the mean over all datasets.

The reproducibility of the FARO Edge measured coordinates was examined by computing the standard deviation (SD) from the mean for each measurement point — both tips of every seed marker in the seed assembly pieces. FARO Edge measured coordinates were reproducible from dataset to dataset with a mean SD of 0.12 mm for the X and Y coordinates, and 0.08 mm for the Z coordinates. The x‐ and y‐axes were considered together due to the fact that, for each seed ring, they fall into the same measurement plane and, therefore, exhibit the same behavior. That is, if the seed assembly piece mounted on the table for measurement were to be rotated 90°, a Y coordinate would become an X coordinate and vice versa. Given that the mounting rotation has no bearing on the final outcome, distinguishing between the two in an analysis of the reproducibility was deemed to be unnecessary. Histograms showing the distribution of SDs for the x‐ and y‐ and z‐axes are shown in Figs. 5(a) and (b).

**Table 2:** Measured seed end coordinates for 10, 18, 20, and 24 mm plaques with respective deviations from the modified TG‐129 or specification coordinates, rendered in the TG‐129 coordinate system, as illustrated in Fig. 2

	*Seed End Coordinates (mm)*						*Deviation (mm)*		
*Seed #*	$x_{1}$	$y_{1}$	$z_{1}$	$x_{2}$	$y_{2}$	$z_{2}$	$\Delta_{1}$	$\Delta_{2}$	*TG‐129 Seed #*
*10 mm plaque*									
1	2.51	−0.01	−2.43	−2.69	0.01	−2.42	0.04	0.15	5
2	4.10	0.54	−1.93	0.50	4.27	−2.14	0.16	0.18	3
3	−0.41	4.12	−1.92	−4.13	0.37	−2.05	0.10	0.08	4
4	−4.13	−0.38	−1.92	−0.34	−4.22	−2.00	0.11	0.10	1
5	0.39	−4.19	−2.07	4.20	−0.52	−1.99	0.07	0.10	2
*18 mm plaque*									
1	2.51	−0.11	−2.46	−2.49	−0.04	−2.53	0.19	0.22	21
2	2.54	1.93	−2.37	−2.58	1.95	−2.26	0.17	0.09	20
3	−2.50	−2.11	−2.40	2.53	−2.03	−2.31	0.24	0.14	19
4	4.88	1.35	−1.52	1.39	5.02	−1.61	0.22	0.10	17
5	−1.36	4.91	−1.62	−5.04	1.36	−1.56	0.16	0.10	18
6	−4.90	−1.46	−1.58	−1.40	−5.01	−1.61	0.23	0.11	15
7	1.34	−5.16	−1.66	5.03	−1.39	−1.63	0.11	0.09	16
8	6.06	−2.43	−0.97	5.96	2.53	−0.99	0.26	0.28	12
9	5.16	3.94	−1.06	0.87	6.55	−0.99	0.30	0.17	13
10	−0.94	6.44	−0.97	−5.10	4.12	−0.94	0.29	0.31	14
11	−6.02	2.47	−1.00	−5.98	−2.57	−1.06	0.27	0.27	9
12	−5.03	−4.01	−0.97	−0.70	−6.74	−1.01	0.38	0.15	10
13	0.98	−6.51	−1.00	5.24	−3.94	−0.99	0.26	0.20	11
14	7.49	−2.48	0.02	7.66	2.42	0.05	0.27	0.24	5
15	7.12	3.60	0.02	3.77	7.19	0.07	0.21	0.26	6
16	2.40	7.68	0.08	−2.47	7.72	0.11	0.27	0.23	7
17	−3.55	7.38	0.01	−7.40	3.69	0.02	0.08	0.15	8
18	−7.62	2.65	0.06	−7.65	−2.50	0.06	0.12	0.18	1
19	−7.30	−3.53	−0.06	−3.69	−7.18	0.10	0.05	0.22	2
20	−2.50	−7.49	0.05	2.41	−7.69	0.03	0.27	0.25	3
21	3.74	−6.86	0.04	7.12	−3.62	−0.03	0.49	0.21	4
*20 mm plaque*									
1	2.52	0.13	−2.36	−2.43	0.03	−2.34	0.19	0.23	24
2	2.39	2.12	−2.07	−2.44	2.16	−2.19	0.33	0.23	23
3	−2.29	−2.17	−2.23	2.43	−2.22	−2.21	0.37	0.23	22
4	4.52	−2.68	−1.75	4.45	2.29	−1.54	0.26	0.44	19
5	3.66	3.54	−1.63	−0.81	5.15	−1.58	0.34	0.29	20
6	−2.22	4.65	−1.60	−4.95	0.72	−1.68	0.26	0.44	21
7	−4.92	−0.73	−1.58	−2.15	−4.51	−1.66	0.45	0.42	17
8	−0.90	−4.96	−1.59	3.74	−3.61	−1.55	0.38	0.24	18
9	6.50	2.10	−0.84	3.52	5.85	−0.73	0.43	0.41	14
10	2.30	6.54	−0.67	−2.35	6.52	−0.69	0.39	0.35	15
11	−3.51	6.01	−0.68	−6.48	2.28	−0.72	0.25	0.45	16
12	−6.77	1.13	−0.75	−5.69	−3.77	−0.78	0.36	0.42	10
13	−5.04	−4.55	−0.70	−0.72	−6.87	−0.68	0.43	0.38	11
14	0.56	−6.90	−0.59	4.92	−4.91	−0.64	0.29	0.38	12
15	5.58	−4.24	−0.82	6.83	0.68	−0.69	0.43	0.51	13
16	8.42	−2.72	0.55	8.50	2.11	0.68	0.21	0.55	5
17	7.95	3.87	0.68	4.86	7.46	0.74	0.51	0.14	6
18	3.62	8.15	0.74	−1.07	8.81	0.70	0.52	0.15	7
19	−2.44	8.60	0.76	−6.63	6.08	0.58	0.48	0.07	8
20	−7.15	5.23	0.75	−8.71	0.44	0.62	0.24	0.28	9
21	−8.74	−0.55	0.74	−7.07	−5.26	0.74	0.29	0.22	1
22	−6.14	−6.08	0.72	−2.06	−8.48	0.72	0.46	0.31	2
23	−0.84	−8.64	0.69	4.01	−7.90	0.70	0.41	0.16	3
24	5.06	−7.37	0.80	8.19	−3.55	0.65	0.31	0.11	4
*24 mm plaque*									
1	2.44	−0.04	−2.30	−2.33	0.05	−2.33	0.23	0.33	
2	2.35	2.03	−2.15	−2.30	2.11	−2.13	0.33	0.41	
3	−2.31	−2.12	−2.16	2.22	−2.09	−2.22	0.40	0.45	
4	4.70	−1.47	−1.71	3.66	3.08	−1.71	0.36	0.46	
5	2.64	4.05	−1.72	−1.97	4.57	−1.69	0.56	0.25	
6	−2.97	3.74	−1.68	−4.90	−0.61	−1.70	0.56	0.32	
7	−4.67	−1.70	−1.67	−0.90	−4.92	−1.65	0.40	0.29	
8	0.13	−4.91	−1.66	4.16	−2.76	−1.58	0.41	0.36	
9	6.14	2.76	−0.91	2.80	6.13	−0.81	0.42	0.27	
10	1.76	6.61	−0.73	−2.95	5.99	−0.80	0.28	0.36	
11	−3.95	5.30	−0.88	−6.58	1.34	−0.97	0.47	0.35	
12	−6.75	0.18	−0.82	−5.19	−4.28	−0.82	0.38	0.30	
13	−4.46	−5.11	−0.72	0.14	−6.81	−0.83	0.27	0.21	
14	1.36	−6.64	−0.83	5.36	−4.23	−0.78	0.39	0.26	
15	5.94	−3.08	−0.86	6.50	1.55	−0.76	0.41	0.35	
16	8.28	−1.65	0.37	7.90	3.02	0.42	0.33	0.36	
17	7.36	4.13	0.45	4.18	7.40	0.51	0.40	0.40	
18	3.10	7.99	0.47	−1.49	8.45	0.52	0.27	0.44	
19	−2.70	8.20	0.43	−6.59	5.39	0.37	0.16	0.36	
20	−7.29	4.50	0.46	−8.33	−0.05	0.42	0.21	0.51	
21	−8.26	−1.10	0.31	−6.39	−5.43	0.39	0.37	0.47	
22	−5.67	−6.44	0.41	−1.39	−8.45	0.45	0.24	0.35	
23	−0.16	−8.58	0.33	4.36	−7.30	0.34	0.29	0.35	
24	5.18	−6.50	0.24	7.95	−2.91	0.42	0.45	0.49	
25	10.07	2.23	2.09	7.79	6.62	2.14	0.27	0.26	
26	6.02	8.23	2.12	1.89	10.04	2.14	0.53	0.35	
27	−0.46	10.35	2.07	−4.90	9.07	2.26	0.25	0.47	
28	−6.86	7.61	2.18	−9.55	3.64	2.02	0.27	0.39	
29	−10.14	1.34	2.28	−9.72	−3.41	2.22	0.36	0.31	
30	−8.63	−5.52	2.16	−5.28	−8.76	2.12	0.33	0.40	
31	−2.94	−9.81	2.15	1.59	−10.08	2.18	0.43	0.42	
32	3.88	−9.43	2.11	7.64	−6.67	2.08	0.34	0.47	
33	9.22	−4.68	2.24	10.29	−0.13	2.28	0.36	0.34	

## IV. CONCLUSIONS

The match between the modified TG‐129 coordinates and the coordinates determined by use of the FARO Edge show excellent agreement, as do the specification coordinates for the 24 mm silastic seed assembly and its corresponding measured coordinates. Seed midpoint coordinates are confirmed to be accurately documented within TG‐129 for the 10, 18, and 20 mm plaques. It may be prudent for clinical physicists to account for the 5.3 mm seed slot length in the silastic inserts if interested in the seed tip coordinates. The FARO Edge has demonstrated itself to be a useful new tool in determining seed coordinates for the 10, 18, and 20 mm COMS silastic inserts and, therefore, lends confidence to the establishment of the specification coordinates for the 24 mm silastic mold as the standard 24 mm COMS‐like eye plaque seed coordinates. These coordinates could be used as input to a Monte Carlo calculation to determine the dosimetry for the 24 mm plaque.

## ACKNOWLEDGMENTS

The authors would like to recognize the help of the Mayo Division of Engineering, particularly from Tom Christensen for his work on the 24 mm and original COMS molds, and Terry Reed and Tyler King. Additionally, the assistance and cooperation of the owner and staff of Trachsel Dental Studio was crucial in being able to complete this project. We also wish to thank Dr. Robert Kline, whose recollections and explanations were enlightening.
